# Multidisciplinary Application of Robotic Surgery in Cancer Disease

**DOI:** 10.3390/cancers15204937

**Published:** 2023-10-11

**Authors:** Jens Hoeppner, Michael Thomaschewski

**Affiliations:** 1 Department of Surgery, University Medical Center Schleswig-Holstein, Campus Lübeck, Ratzeburger Allee 160, 23538 Lübeck, Germany; michael.thomaschewski@uksh.de; 2 Faculty of Medicine, Albert-Ludwigs-University of Freiburg, 79085 Freiburg, Germany

Robotic assistance systems are utilized in minimally invasive surgery with a rapidly increasing frequency. The possible uses are wide and robotic assistance systems are routinely used by various surgical disciplines. A clear focus of application in many disciplines is surgical oncology. The goal of this Special Issue of Cancers is to provide an overview of the possibilities and development of multidisciplinary application of robotic surgery in surgical oncology. It is based on reviews and research articles that report emerging evidence of robotic cancer surgery in colorectal, prostate, kidney, and lung cancer. Minimally invasive surgical techniques for the surgical treatment of tumor diseases of many visceral organs have been developed in recent decades and have significantly improved patient outcomes. The most significant current technical development in this field is the supplementation of minimally invasive techniques with robotic assistance systems. This sustainably improves intraoperative visualization, surgical instrument control, and training possibilies.

A key subject of clinical research is the comparison of conventional minimally invasive surgical techniques with newly developed techniques that utilize robotic assistance systems. Against this background, Tschann et al. compared operative endpoints and postoperative results in the treatment of colorectal cancer in a propensity score-matched analysis [1]. The main result of this analysis was that the probability of conversion to an open surgical procedure was significantly reduced in the robot-operated patient group (*n* = 16 (19.51%) vs. *n* = 5 (5.38%), *p* = 0.004). The postoperative morbidity and oncological surrogate parameters, however, were equivalent for both patient groups examined [1]. Another retrospective comparative study in lung cancer compared the conventional minimally invasive technique (VATS) with the robot-assisted (RATS) technique in a collective of 844 patients [2]. The study hypothesized that the technical advantages of RATS, compared with VATS, assure more precise tumor resection and advancement in radical lymph node dissection, which should increase oncological outcomes compared to VATS. Concerning the chosen endpoints of 5 years and 3 years of disease-free survival, the study failed to show significant differences in lung lobectomy and segmentectomy between VATS and RATS [2].

An important subject of research is the analysis of the teaching and transfer of robotic surgical techniques, along with the study of learning curves in the acquisition of the techniques. In the context of rectal cancer, a retrospective study was conducted on 146 patients to compare the learning curves between transanal total mesorectal excision (taTME) and transabdominal robot-assisted rectal resection. The comparison was biased as patients undergoing taTME had fewer lesions and were more likely to be men and to receive neoadjuvant treatment. In the study, the morbidity rate started to decrease after the 17th case of taTME and after the 49th case of robot-assisted surgery. In the initial learning phase, the rates of anastomotic leakage (35.7% vs. 5.7%) and urethral injuries were higher in taTME. However, the conversion rate was higher in robot-assisted surgery (1.5% vs. 10.1%) [3]. Another study on prostate cancer examined the transferability of the quality of results of robotic prostatectomy from an established specialized treatment center to a newly established treatment center without previous team experience in robotic surgery [4]. The procedures were performed by the same surgeon during consecutive treatment periods (May 2018–December 2018 vs. December 2018–December 2019). The study compared tumor characteristics and surgical intraoperative and pathological result parameters as endpoints. In the newly established center, the duration of surgery (149 vs. 172 min, *p* < 0.01) was shorter and blood loss was lower (300 vs. 131 mL, *p* < 0.01). No difference was established in the proportion of positive surgical margins in the new center (8.8% vs. 8.2%, *p* = 1.00) [4].

Another important research direction is the further development of the surgical instruments used in robotic surgery. Since robot-assisted surgery is a very recent development in the surgical field, the selection of instruments is relatively limited. The transfer of surgical techniques to the context of robotic surgery also largely requires the development of new surgical instruments. An example of this is the study by Gabi et al., which describes the preclinical development of a tissue-holding instrument (THD) for robot-assisted minimally invasive partial nephrectomy in kidney cancer [5]. THD is a vacuum-based device composed of 3D-printed polyethylene or stainless steel. The article reports feasibility studies performed on porcine kidneys, porcine livers, and human cadavers. In porcine tissue, the device setup, tissue suction, and handling were positively evaluated. In a simulated transabdominal approach in human cadavers, the device setup, suction, and tissue handling were also rated positively. No compromising effects of macroscopic tissue damage were found [5].

Robotic operations occur in a multidisciplinary medical environment and, in addition to surgical factors, strongly influence perioperative parameters. In particular, the anesthesiologic environment, perioperative monitoring, and organ function control that occur in this context are also affected. An exciting example of multidisciplinary research in this field is a prospective randomized double-blind controlled trial on 80 patients with kidney cancer comparing an intraoperative bundle strategy consisting of remote ischemic preconditioning (RIPC) and an intrathecal morphine block (ITMB) versus routine treatment without RIPC and ITMB in robot-assisted laparoscopic partial nephrectomy [6]. The primary outcome was of the trial postoperative kidney function, defined as the lowest estimated glomerular filtration rate (eGFR) on postoperative day 2. The secondary endpoints were defined as surgical complications, pain, and length of hospital stay. Although the primary endpoint was negative, the non-bundle group had longer hospital stays and more severe pain than the bundle group. Overall, no severe surgical complications were observed in either group in the trial [6].
